# Mean Knowledge Score of Self-Medication among First and Second Year Medical and Dental Students in a Medical College: A Descriptive Cross-sectional Study

**DOI:** 10.31729/jnma.6443

**Published:** 2021-11-30

**Authors:** Sajala Kafle, Nisha Jha, Pathiyil Ravi Shankar

**Affiliations:** 1 Department of Pharmacology, KIST Medical College, Lalitpur, Nepal; 2 IMU Centre for Education, International Medical University, Kuala Lumpur, Malaysia

**Keywords:** *anti-bacterial agents*, *cross-sectional studies*, *Nepal*, *self-medication*, *students, dental*, *students, medical*

## Abstract

**Introduction:**

Self-medication is common among medical students. Medical and dental students can also educate the population about the use of medicines. The aim of the study was to find the mean knowledge score of self-medication among first and second year medical and dental students at a medical college in Nepal.

**Methods:**

A descriptive cross-sectional study was carried out in a medical college from 1st to 28th February 2021 using a structured online questionnaire after obtaining approval from the Institutional Review Committee (approval number 077/78/32). Convenience sampling method was undertaken. The responses were analyzed using Statistical Package for Social Sciences version 26.

**Results:**

Out of 173 students the mean±SD knowledge score was 79.47±6.76. The mean knowledge score of the participants of age group 17-18, 19-20 and 21-22 years were 83.8, 79 and 79.1 respectively. The mean knowledge scores of the male and female participants were 80.2 and 78.6 respectively. Similarly, the knowledge scores of respondents from undergraduate medical and dental backgrounds were 80.1 and 77.7 respectively.

**Conclusions:**

Knowledge of self-medication was high among the students. However, educational sessions to further improve and strengthen knowledge can be carried out to improve the knowledge.

## INTRODUCTION

Self-medication is defined as the selection and use of medicines to treat self-recognized illness or symptoms without consulting a healthcare professional.^1,2^ Inappropriate use of medicines as self medication can have harmful effects on humans causing wastage of resources and increasing resistance to pathogens.^3-6^ Severe adverse drug reactions and drug dependence can also occur.^3,7,8^ The use of different drugs for self medication is very common in developing countries.^3,9-12^

Medical and dental students are more likely to self-medicate as they are knowledgeable about diseases and drugs. In addition, they also have easy access to drug information in the literature.^13^ Medical and dental students are future drug prescribers. Hence, they should have adequate knowledge and proper attitude toward self-medication.

The aim of the study was to find the mean knowledge score of self-medication among first and second year medical and dental students at a medical college in Nepal.

## METHODS

A descriptive cross-sectional study was conducted among first year and second year medical and dental students at KIST Medical College and Teaching Hospital from 1^st^ to 28^th^ February 2021. The ethical approval was obtained from the Institutional Review committee of KIST Medical College and Teaching Hospital (approval number 077/78/32). All medical and dental students from first year and second year who provided consent to participate were included in the study. Students who did not give consent and those from other years of study were excluded from the study. Convenience sampling method was used and the sample size was calculated using the formula,

n = Z^2^ × σ^2^ / e^2^

  = (2.32)^2^ × (0.5)^2^ / (0.1)^2^

  = 135

where,

n= required sample size,
Z= 2.32 at 98% Confidence Interval (CI)
σ= standard deviation, 0.5
e= margin of error, 0.1

Adding 10% non respondents rate the new sample size becomes 165. However we took a sample of 173 students. The questionnaire was pretested and then distributed to class representatives through social networking sites. The class representative forwarded the link to their classmates. A previously validated questionnaire was used in this study to evaluate knowledge among the respondents.^14^

The questionnaire in this study had two sections. Respondents completed the questionnaire after providing consent online. The first section contained the demographic details. Second section contained twenty statements for evaluating the participant's knowledge. Maximum scores for knowledge were 100. A score more than 50% was considered as an adequate score and less than 50% was considered as inadequate.

The responses were analyzed using Statistical Package for Social Sciences version 26 for Windows. The demographic parameters were tabulated and the knowledge of medical and dental students regarding self-medication evaluated.

## RESULTS

Out of 173 students the mean±SD knowledge score was 79.47±6.76. The mean knowledge score of the participants of age group 17-18, 19-20 and 21-22 years were 83.8, 79 and 79.1 respectively. In addition to this, the mean knowledge scores for participants from the rural and urban area were 77.8 and 80.2 respectively. Furthermore, the mean knowledge scores of the participants whose mothers were related to the medical field, not related to the medical field were and housewives were 83.1, 80.9 and 78.5 respectively (Table 1).

**Table 1:** Mean knowledge scores among different subgroups of respondents (n =173).

Characteristics	Mean Knowledge score (SD)
**Age**	
17-18	83.8±7.9
19-20	83.8±7.9
21-22	79±6.4
Family home	
Rural	77.8±6.5
Urban	80.2±6.7
Profession of Mother	
Related to medical field	83.1±5.5
Not related to medical field	80.9±7.0
Housewife	78.5±6.5

Out of the total 173 participants, 83 (43%) participants were in the age group of 19-21 years. Ninety-one (52.6%) were males and 82 (47.4%) were females. Out of 173 participants, 127 (73.4%) were medical (MBBS) and 46 (26.6%) were dental (BDS) students. Majority of the participants 116 (67.1%) were from urban areas. Among the 173 participants, only 10 (5.8%) had a father's profession related to the medical field and 115 (66.5%) participants' mother was a housewife (Table 2).

**Table 2:** Demographic characteristics of respondents (n =173)

Characteristic	n (%)
Age (in years)	
17-19	14 (8.1)
19-21	76 (43.9)
21-23	83 (48)
Gender	
Male	91 (52.6)
Female	82 (47.4)
Education	
MBBS	127 (73.4)
BDS	46 (26.6)
Year of study	
MBBS First year	78 (45.08)
MBBS Second year	63 (36.41)
BDS first year	18 (10.4)
BDS second year	14 (8.09)
Family home	
Rural	57 (32.9)
Urban	116 (67.1)
Profession of Father	
Related to medical field	10 (5.8)
Not related to medical field	163 (94.2)
Profession of Mother	
Related to medical field	8(4.6)
Not related to medical field	50 (28.9)
Housewife	115 (66.5)

## DISCUSSION

The present study was undertaken among the first and second year medical and dental students. The knowledge score was adequate among the respondents as we had regarded a score of more than 50% as adequate. The score of 79.47% can even be regarded as good to high.

The demographics of the respondents in the current study is different from another study conducted in Karachi were 41.1% of respondents were male and 58.9% were female.^15^ The total percentage of students from first year was 55.49% and total percentage of students from second year was 44.5%. In our study, the participants between the age group of 17 to 19 years had the highest mean knowledge score of 83.8%. There are various reasons for self-medication. Some of these are easy availability of medicines, socioeconomic factors and lack of time and previous experience of symptomatic relief with the drug.^16,17,18^ This is like another study conducted in Nepal where the mean knowledge score of the medical students from both basic sciences and clinical years was 74.56%.^19^ In another study conducted in Nepal, the mean knowledge score was 74.54%. Also, in the same study, no significant difference in knowledge scores according to respondents' age was found.^14^ However, in another study it was shown that participants had poor knowledge about self-medication, but selfmedication was commonly practiced.^3^

Study participants from urban areas had significantly higher mean knowledge scores than the participants from rural areas. This finding is also different than another study conducted in Nepal where no significant difference in knowledge scores according to the address of the participants was found.^14^

Family characteristics may also influence the knowledge and practice of self-medication.^3^ Studies have shown that mothers' education and profession also increase the chances of self-medication among adolescents.^20^ Regarding the knowledge scores according to the profession of mother, those participants whose mother was related to the medical field had higher knowledge scores as compared to the participants whose mother were from other professions. This finding is also different compared to another study conducted in Nepal where no significant difference in knowledge scores according to the profession of mother was noted.^14^

The major limitation of the study is that it was conducted in only one medical college of Nepal. Also, this study included only the medical and dental students from first year and second year. Convenience sampling was done and only 72% of the student population participated.

## CONCLUSIONS

Students have higher knowledge about self-medication than in other similar studies. The students between the age group of 17 to 18 years had the highest total knowledge score. Educational sessions to further strengthen their knowledge can be considered.
